# Interpersonal, institutional, and structural racism in Canadian nursing: A culture of silence

**DOI:** 10.1177/08445621221110140

**Published:** 2022-06-23

**Authors:** Brenda L. Beagan, Stephanie R. Bizzeth, Josephine Etowa

**Affiliations:** 1 School of Occupational Therapy, 70338 Dalhousie University, Halifax, NS, Canada; 2 60476 Dartmouth General Hospital, Dartmouth, NS, Canada; 3 Black Women’s HIV Prevention and Care, Faculty of Health Sciences, 70363 University of Ottawa, Ottawa, ON, Canada

**Keywords:** Racism, whiteness, nurse education, nursing practice, qualitative approaches

## Abstract

**Background:**

Alongside declarations against racism, the nursing profession in Canada needs examination of experiences of racism within its ranks. Racism at multiple levels can create a context wherein racialized nurses experience barriers and ongoing marginalization.

**Purpose:**

This critical interpretive qualitative study asks how interpersonal, institutional, and structural racisms intersect in the professional experiences of racialized nurses in Canada, and how nurses respond.

**Methods:**

Self-identified racialized nurses (n = 13) from across Canada were recruited primarily through snowball sampling, and each was interviewed by phone or in person. Once transcribed, interviews were analyzed inductively, which led to the levels of racism as a guiding framework.

**Results:**

From entry to nursing education throughout their careers participants experienced racism from instructors, patients, colleagues and managers. Interpersonal racism included comments and actions from patients, but more significantly lack of support from colleagues and managers, and sometimes overt exclusion. Institutional racism included extra scrutiny, heavier workloads, and absence in leadership roles. Structural racism included prevalent assumptions of incompetence, which were countered through extra work, invisibility and hyper-visibility, and expectations of assimilation. Racialized nurses were left to choose among silence, resisting (often at personal cost), assimilation and/or bolstering their credibility through education or extra work. Building community was a key survival strategy.

**Conclusions:**

Everyone in nursing needs to challenge the culture of silence regarding racism. White nurses in particular need to welcome discomfort, listen and learn about racism, then speak out to help disrupt its normative status.

## Background & purpose

Reflecting on racism in Canadian health professions, Dhara (2020) writes:

*The most damaging feeling is an unrelenting message that I don't belong. I am not white, and as such, I am asked incessantly to explain my brown skin. The questions range from shocking to snide. I am asked where I am from, where I graduated, if I know that Muslim person in town, why my English is “perfect,” whether I speak Hindu, what I think about honour killings, where I do yoga, whether I plan to stay in Canada, and the best Indian restaurants, stores, cookbooks, movies, and vacations. The truth is that it doesn't matter whether people mean well. The effect is that I am repeatedly made aware that my position both as a physician and a Canadian is tenuous at best*. (p. 596)

This depiction captures the way everyday interpersonal interactions can embody systemic racism, the way structures of white supremacy, or institutionalized whiteness, are instantiated in relentless ‘innocuous’ verbal assaults known as ‘microaggressions’ (Sue, 2010). It captures the intersections of structural, institutional, and interpersonal racism. In a nation constructed through colonial race relations, it would be surprising to find any institution not infused with racism, a system of oppression foundational to the sociopolitical economy of Canada. Racism affects health care from recruitment into the professions, through professional education, into the structures and everyday interactions of practice, to ultimately affect health care delivery. Racism within health care is one of the ways it operates as a social determinant of health.

In recent years the Canadian Nurses Association released a declaration against anti-Indigenous racism (CNA, 2021a) as well as a statement declaring anti-Black racism a public health emergency in Canada (CNA, 2020; see also CNA, 2021b). Racism *within* the profession is given only passing mention. As Roger Kline (2020) argued in the context of the UK's National Health Service, “Strong statements on racism are helpful. But in 2020 anything less than decisive practical action is unforgivable.” With an action orientation, around the same time a Black Nurses Task Force was formed by the Registered Nurses Association of Ontario to tackle anti-Black racism within the profession (https://rnao.ca/content/rnaos-black-nurses-task-force), and a group entitled the Canadian Black Nurses Alliance was formed to challenge racism within and provide peer support (Onagbeboma, 2020).

Critical analysis of specific professional contexts can explicate the processes through which racism is integrated into routine everyday practice, helping to identify avenues for change. This paper examines the professional experiences of 13 racialized nurses in Canada, examining how structural racism infuses institutional and interpersonal practices, and how coping and resistance strategies are themselves complicated by racism. We use the term ‘racialized groups’ to signal that ‘race’ is not a natural or biologically-meaningful concept, rather some groups are marked as subordinate through sociopolitical and historical process of categorizing groups hierarchically (Miles, 1989). In other words, racialized groups are created through social processes of racism. In Canada this includes anti-Black racism, but also racism against other nonwhite groups.

### Conceptualizing racism

In this paper we employ the framework of interpersonal, institutional and structural racism delineated by Nazroo et al. (2020). Their framework is particularly helpful in its attention to micro, meso and macro levels, and the important interconnections across levels. At the micro level, interpersonal racism concerns interactions between individuals, the everyday looks, jokes, questions and comments, as well as the prejudices, discrimination and even violence that occur routinely (Essed, 1991). It includes microaggressions, the commonplace racialized questions and comments that – intentionally or not – communicate messages of Othering, conveying difference and subordination (Sue, 2010). These cumulatively add to an exhausting burden. Interpersonal racism can be vicarious, such as witnessing or overhearing the targeting of another racialized person, knowing it could well have been you (Essed, 1991). It draws its power from broader structural racism, without which it would simply be ill-will or poor behavior. At the same time, institutional racism often acts through interpersonal encounters, as institutions operate through persons.

At the meso level, institutional racism involves (re)producing racism through the practices, policies, and procedures of organizational operations (Nazroo et al., 2020). Institutional features like standards, rules, expectations and operating processes are ostensibly race-neutral, yet systematically advantage white people while excluding or disadvantaging people of color (Crenshaw et al., 1995). Institutional practices are enacted by individuals, yet they also comprise the distilling or concentrating of structural racism into routine practices. The disembodied practices of institutions may be very challenging to alter.

At the macro level, structural racism refers to patterned social arrangements that result in inequitable access to and advantage from the financial, material, political, symbolic and social resources of a society (Nazroo et al., 2020). While some position cultural and ideological racism as distinct forms of racism, in this framework structural racism includes cultural and ideological dimensions, which support and justify inequities. For example, values like ‘meritocracy,’ achieving on the basis of individual merit with no socially-structured advantage, are foundational to structural racism in Canada. Beliefs about what counts as expertise or authority are shaped by and in turn perpetuate structural racism, establishing white ways of knowing and doing as legitimate and authoritative while systematically silencing and delegitimizing other knowledges and values (Dotson, 2011; Mills, 2007). Structural racism pertains to the social structures of a society, the ways inter-connected social arenas (e.g., media, education, health care, policing, legal system, politics) operate to produce racialized hierarchies.

In short, racism is not about random acts by lone individuals, it is built into ostensibly ‘neutral’ institutions and social structures (Cabrera et al., 2016), manifested in policies, procedures, belief systems and interpersonal interactions. White supremacy and systemic racism are normative, the *status quo* of institutions and social arrangements in Canada.

### Racism in nursing

As a profession, nursing is not immune to systemic racism. Modibo (2004) documents virulent racist attacks against Black nurses in Ontario in the 1990s, which some term ‘racist bullying’ (Allan et al., 2009). More common today are microaggressions conveying messages of not-belonging (Vukic et al., 2012). Such incidents may involve coworkers, managers, other staff and/or patients (Cottingham et al., 2018; Etowa et al., 2009; Iheduru-Anderson, 2020). About being racialized and Muslim in Canada nursing, Saleh (2017) comments, “Many times, I am reminded — through someone's casual comment, a momentary stare or an intentional othering (‘not one of us’) statement — that I am different and that I do not belong” (p. 34). The interpersonal racism intensifies when white colleagues, bystanders, do and say nothing. Silence and inaction in response to interpersonal racism is a form of complicity, helping maintain systemic racism, and communicating to racialized nurses their isolation and lack of value (Bouabdillah et al., 2021; Etowa et al., 2009; Iheduru-Anderson et al., 2021). This leaves racialized nurses to invest time and energy (that might otherwise go to patient care) into managing racist microaggressions.

Resistance to the authority of racialized nurses in leadership roles (Bouabdillah et al., 2021; Jefferies et al., 2018; Onagbeboma, 2020) is form of interpersonal racism that illustrates the intersections of interpersonal, institutional and structural racism. When individual nurses resist racialized leadership, this both draws on and feeds cultural ideologies that construct leadership in terms consistent with whiteness. It reiterates beliefs regarding the ‘natural’ inferiority of racialized peoples (Brathwaite, 2018). Institutionally, at the level of the profession as well as individual workplaces, racialized nurses are vastly under-represented in leadership and management positions, disproportionately located in lower levels of the profession (Bouabdillah et al., 2021; Calliste, 1996; Das Gupta, 1996; Jefferies et al., 2018; Premji & Etowa, 2014).

Institutionalized racism also plays out through workload allocation, with racialized nurses disproportionately relegated to the ‘heaviest’ units, such as acute and chronic care, while relatively absent in specialty units (Das Gupta, 1996; Modibo, 2004). They may be subject to higher levels of surveillance and discipline (Calliste, 1996; Etowa et al., 2009; Modibo, 2004). In turn, racialized nurses may find they have to “work harder, be smarter, and prove themselves more than their White peers” (Iheduru-Anderson et al., 2021, p. 120). In one Canadian study, Black nurses showed “an exaggerated degree of commitment, by desiring to be a perfect nurse or a super nurse in an effort to counter criticism” (Etowa et al., 2009, p. 177). As one nurse said, “I made sure to cross all my Ts and dot all my Is very carefully, always looking over my back a little bit… You want to prove to everybody that you are capable, and more than capable” (p. 177). This was echoed almost literally by a nurse in a US study, about a decade later: “I got to cover my back, make sure I got everything- all i's dotted, t's crossed, because someone is gonna check behind me, doubt me, say that I’m wrong. It happens over and over and over again” (Cottingham et al., 2018, p. 153).

The extra work required of racialized nurses (managing heavier workloads, proving themselves super-capable) is coupled with extra emotional labor, work required “to remain and succeed in white institutions” (Cottingham et al., 2018, p. 145). While all nurses must manage emotions, particularly when working with ‘difficult’ patients, racialized nurses must deal with the additional onslaught of racism from patients and coworkers. For example, when patients are in physical or psychic pain they may become aggressive with any nurse, using whatever resources are at hand; the ubiquity of racism leaves racist slurs readily available as a cultural resource with which to lash out at racialized nurses (e.g., Saleh, 2017). The work of managing the pain of such overt racism – excused because of patient illness, yet manifesting prevalent beliefs and perceptions – is exhausting and demoralizing (Cottingham et al., 2018). So too, the additional work of constantly educating coworkers, constantly having to decide whether/when to speak up, is exhausting (Etowa et al., 2009).

Finally, structural racism intersects with interpersonal and institutional racism by supplying the beliefs and prejudices, the assumptions built into routine practices, and the justifications for racial inequities as being about anything other than racism. Whiteness pervades nursing, starting in education, where curricula are decidedly Eurocentric (English, 2021; Garland & Batty, 2021; Jefferies et al., 2018; Onagbeboma, 2020; Prendergast et al., 2020). It extends into nursing research and inquiry, which are shaped by epistemological commitments to whiteness (Hilario et al., 2018; Sinclaire et al., 2021). As Brathwaite (2018) suggests, the entrenched belief in the inferiority of racialized nurses is a legacy of colonial racism.

In this paper we draw on the commitment of Critical Race Theory to counter-storytelling in order to surface subordinated realities (Crenshaw et al., 1995), we ask, How do institutional, structural and interpersonal racisms intersect in the professional experiences of racialized nurses in Canada, and how do they resist and respond? While explicating racism within the profession will not be ‘news’ to racialized nurses, in the face of prominent professional declarations of commitment to anti-racism, we offer this analysis as a corrective to the continuing silence regarding racism *within* the profession, contributing to an epistemic disruption of the dominant discourse of a caring profession (Cottingham et al., 2018; Hilario et al., 2018).

## Methods and procedures

This paper draws on a subset of data from a larger study examining the experiences of health professionals (physicians, nurses, occupational therapists) who self-identify as disabled, working class origin, racialized, ethnic minority, and/or minority sexual/gender identity. It does not include the experiences of Indigenous professionals, which will be a second phase of the research. The study was approved by three university research ethics boards.

Nurses with at least five years Canadian practice experience were recruited through team members’ professional networks, snowballing, and dissemination of recruitment information through professional newsletters. Those who responded were emailed consent information, eligibility was confirmed, and in-person or telephone interviews (60–90 min) were scheduled, typically outside of work hours. From the interviews conducted with 49 participants (including nurses, physicians and occupational therapists) across Canada, this analysis draws on transcripts solely from the 13 nurses who self-identified as racialized.

The study was grounded in critical phenomenology, in the sense that it focused on the taken-for-granted aspects of everyday life, with a critical attention to power relations that structure lived experience (Ahmed, 2006). After discussing consent, qualitative interviews explored belonging and marginality, the toll of oppression, as well as coping and resistance. Interview questions were sent in advance only if requested, since they served more as a guide than a script. Interviews were conducted by one of three interviewers who were all members of the marginalized groups in the larger study, and who were health professionals and/or social scientists, with PhDs in progress or completed. Interview recordings were transcribed verbatim, then coded using ATLAS.ti software. Participants received an online gift card as an honorarium.

Iterative analysis moved between compiling coded data (quotations) and re-reading full transcripts, for within-case and cross-case analyses. Analysis moved from data to theory and back again, focusing on codes such as microaggressions, overt hostility, belonging, and coping strategies. Data analysis and team discussions identified the conceptual framework above as best-suited to the data, providing an overarching thematic structure. Quotations were organized and reorganized as sub-themes emerged, then ‘cleaned’ by removing false starts and filler words like ‘um.’

### Rigour

We chose not to use pseudonyms to avoid the violation of mis-naming and to enhance confidentiality, and we report demographics at fairly high levels of abstraction (Table 1) to reduce identifiability. At the same time, this introduces the problem of over-homogenizing diverse groups, for example, referring to nurses from multiple nations and ethnic groups as ‘South Asian.’ While this is a concern, it was preferable to rendering participants identifiable.

**Table 1.** Participant Demographics.

ID#	Identity	Practice area/type^a^	Region, rural/urban	Age (yrs)	Years in practice
#1	South Asian, Muslim	Mental health	Central Canada, Urban	30–35	5–10
#2	African	Critical care	Central Canada, Urban	35–40	5–10
#3	Caribbean	Mental health	Central Canada, Urban	40–45	10–15
#4	African, Muslim	Mental health	Eastern Canada, Urban	30–35	10–15
#5	Southeast Asian	Acute care, community, pediatrics	Central Canada, Urban	35–40	10–15
#6	South Asian	Mental health, acute care	Central Canada, Urban	35–40	10–15
#7	African	Community	Western Canada, Urban	40–45	15–20
#8	African	Mental health	Central Canada, Urban	45–50	15–20
#9	African	Pediatrics & maternal	Central Canada, Rural	45–50	15–20
#10	South Asian	Pediatrics & maternal, academia	Central Canada, Urban	45–50	20–25
#11	African	General nursing	Central Canada, Urban	45–50	20–25
#12	African Canadian	Community	Eastern Canada, Urban	50–55	30–35
#13	African Canadian & Indigenous	Private, community, long term care	Eastern Canada, Rural	55–60	30–35

^a^ some also held academic positions.

Drawing on our previous experience in research with marginalized professionals, we did not employ member-checking, in order to reduce participant burden. Members of our team all identify with the groups included in the study, which enabled us to test for ‘resonance’ of the emerging analyses. From conceptualization of the study, through interviewing, to data analysis and writing the research was informed by lived experience. We employed collective reflexive analysis, challenging and building on emerging interpretations through weekly meetings, and discussions (Dörfler & Stierand, 2020). For rigour in the current analysis, it was helpful to have both racialized and white researchers involved, as well as nurse and non-nurse researchers.

## Results

All of the 13 participants but one identified as women, and all but two practiced in urban areas, ranging from small to large cities. Participants were primarily African heritage, including both migrants and African Canadians, but also Southeast Asian and South Asian (Table 1). Types of practice varied widely, as did ages and years of practice experience. Participants discussed multiple ways racism posed challenges to their everyday work. Below we describe interpersonal, institutional, and structural racism, as well as harms inflicted and ways people responded.

### Interpersonal racism

All participants had experienced direct interpersonal racism involving patients, colleagues, and managers. Overt racist slurs from patients were common among the African-heritage participants. For example, “The first thing they’ll say, ‘Oh, you Black this’ or ‘You Black that’ or the ‘N’ word or, you know?” One participant explicitly stated that nurses are trained to simply walk away from such situations. One African-heritage participant described an incident where a patient called her racist names like ‘baboon’ and ‘jigaboo’; it escalated into a physical incident. Another participant had patients grab her hijab, trying to pull it off. More indirectly, one participant noted that while she did not think she faced overt racism from patients – apart from her credibility being undermined by her accent – she routinely saw nursing students from her racialized group in tears because of hostile patient comments and patients doubting their competence.

Where interpersonal and institutional racism intersect is in the (lack of) response from colleagues and managers to overtly racist incidents. For example, in the instance above where name-calling became a physical altercation, the participant noted that none of her colleagues or management helped; in fact they vanished:

There was a scuffle and I was calling for help. And nobody wanted to come in… This room was directly outside the nursing station and when I went in there, there was all kinds of people sitting around the nursing station. When I came out, there was nobody there. Everybody had just left and gone elsewhere so they didn't have to help.

When she reported the incident to her supervisor, the response was, “Well it looks like we are just going to have to do something about your colour.”

Another way institutional racism was actualized through interpersonal actions was in workload assignments and (lack of) coworker support. Several participants spoke of above average workloads, and being assigned the most complex patient care. For example, one African-heritage nurse reported, “I would always get the acutely sick patients. I would get the complicated patients. I would get the Black patients. I would get the disgruntled patients, all the difficult patients.” In addition to complex caseloads, some found coworkers unwilling to help: “I worked on a floor where they would give you the heaviest load to begin with, so they give you the heaviest patients, and then they don't want to help you.” More than one person noted that when she asked for assistance, it was not forthcoming, a subtle but particularly effective form of ostracism. At the same time, some participants were asked by superiors to do inappropriate tasks and felt unable to resist, “scared of reprimand.” For example, some were routinely asked to translate for patients from other ethno-linguistic groups.

Another form of interpersonal racism was exclusion from or in social interactions, such as colleagues taking breaks without them, or celebrating the accomplishments of white nurses while ignoring racialized nurses who shared in the work. Some colleagues were overtly racist: “There was one of the nurses who was openly racist. She would mock the new nurses, the immigrants.” Sometimes derogatory comments and mocking intensified when participants began to move upward in their careers: “When I started my Masters, that's when I realized that the hostility was really a thing.”

### Institutional racism

Participants described experiences of institutional racism at all levels of career progression. For some it started even before entering the profession, as racism appeared to influence what supports were available to even identity nursing as a potential field of study. For example, one participant said school guidance counselors offered no support; though she was certain she wanted to become a nurse, she had no idea how to make that happen. Another participant noted the challenges of entering nursing as a first-generation Canadian whose family had no financial resources to support advanced education, since her parents’ professional credentials were not recognized in Canada. She maintained paid employment throughout nursing school.

Several participants observed that once in nursing school racialized students were treated more harshly by instructors. One reported Black students were “targeted” by a professor who grilled them relentlessly: “Just put me in a room and question me and question me nonstop about something, and another thing, and the same thing over and over again.” Others reported similar challenges with clinical instructors:

The preceptor that I had at the time would always give me more [work]. Like, she would always want to basically see if I can swim. So pile on all the work, and see If I would be able to swim. And if I drown, if I would call her – you understand? And I realized that it's a tactic that they use, mainly with Black nurses.

Another Black nurse suggested clinical education is doubly challenging with an instructor who “feels as if you are not going to make a good nurse, because of the color of your skin or whatever.”

Once in the profession, many participants found they were the only racialized nurse in their workplace, perpetuating institutionalized whiteness, the sense that white ways of doing things are natural and inevitable. Participants reported a lack of racialization at all levels of management. For example, one participant stated, “There is a lot of Black people… who are highly educated, but when the promotions come, very, very few– I don't think we even have a Black manager in nursing. No, I have never seen one.” This was echoed by another participant who noted that in large multicultural cities most frontline nurses are racialized, but this disappears moving up the hierarchy:

Especially in big cities like [this], you will find a majority of frontline nurses are coming from ethnic backgrounds, from racial minorities. But when you look at the management, and the layers of management, you will find that their representation is even less than scant. I wouldn't even use the term scant for that. So, extremely rare … in those management positions.

Two participants described unsuccessfully applying for management positions while white colleagues with fewer credentials got promotions. One person was uncertain whether she was denied promotion due to her race, or due to wearing hijab. Across types of workplaces, participants spoke of roadblocks to career progression.

The ability to move up is where I’ve experienced… a lot of hurdles… On multiple occasions I’ve been told ‘it will never be you’. It's hard to know whether that was personal, or whether that was because I was an ethnic minority.

One South Asian participant remarked on the need to create opportunities for oneself, “Because no one is going to open that door for you, unless you happen to be [of] a very privileged upbringing or circumstances.”

### Structural racism

Ideas like ‘create your own opportunities’ are common-place expressions of meritocracy, one of the ideological cornerstones of structural racism. Again, structural racism concerns the socially-patterned ways in which racialized people are subordinated; it is implemented through multiple institutions, and justified through cultural ideas and beliefs. Meritocracy is a key cultural construct, the notion that hard work earns just rewards – therefore lack of reward signifies lack or effort or capability. Some participants argued that they were automatically presumed less-capable, thus had to perform twice as well as others to be considered equally good. Even in school, they had to be extra-prepared, perceiving that they were expected to fail:

You cannot go unprepared, that's basically what it is. Do not give people a chance to say, ‘Oh, wow, you didn't do this so because of that you’re out.’ So you don't give people a chance for anything. It's only prepare yourself for– especially clinicals because those are very, very subjective.

As another participant said, “I make sure I’m well prepared… They feel like because you’re Black, that is why you don't know this, right? Or, because you’re Black, that is why you didn't do it this way.” Even when unspoken, negative stereotypes and assumptions haunted the educational and work lives of participants. Consequently, many participants thought they embodied a level of professional perfectionism that is not required of others to be considered qualified and capable.

Particularly when participants were the only racialized persons at their workplaces, they were required to put in extra effort to counteract stereotypes and assumptions regarding the incompetence of racialized nurses. For example, one Black participant reported that if her work was not executed flawlessly, coworkers would escalate the situation immediately to management; meanwhile white coworkers would cover for one another. She thought there was a campaign intended to construct her as incompetent, which she called racist bullying:

The manager there told the girls to write up anything and everything they think I am doing wrong. So I was written up for things like, ‘Oh, you didn't put your initial here’… If I forget anything… I was being written up and after I was being called into the office about everything, and I said, ‘My God, I was a good nurse before!’

This nurse realized later she probably should have filed a human rights complaint, “But I did not, (laughs). I didn't know any better, I was so new. I was so naïve.”

Participants spoke of racialized nurses being both highly visible and highly invisible, suggesting the whiteness expected in professional contexts leaves racialized participants unexpected and invalidated as well as hyper-visible. For example, a South Asian participant described hyper-visibility as the only racialized nurse present:

Because I am the only person of colour I stand out in ways that are not necessarily positive. So I have to present myself differently. I have to make sure my face is visible at meetings, because when the brown face in the crowd is missing, that's noticeable, *versus* when one white face is missing.

At the same time, however, she was often overlooked completely, such as when input was sought from everyone in a meeting except her: “I also feel like I have to work twice as hard as everybody else, in making sure I’m visible.” She described an incident where everyone in the room was asked to introduce themselves except her and one other person of color; she was forced to ask, “Can the brown girls introduce themselves?”

Hyper-visibility along with doubts about competence left most participants conscious about how they presented themselves in work contexts, attending to dress and demeanor to bolster professional credibility with colleagues: “I always make sure I’m dressed in a suit, when I’m at work, whereas everyone else can wear jeans.”

For participants who were not only racialized, but also from ethnic or cultural minority groups, there was an added element of needing to learn how (whether) to adapt to white, Western cultural norms and expectations implicitly considered superior. Both education and practice assumed familiarity with Eurocentric cultural referents and communication styles. For example, one African-heritage nurse noted that culture-bound expectations had an everyday impact on her work:

Nurses are direct with people, like, make eye contact and everything; I was always scared that I’ll never be able to make eye contact. Because in my ethnicity, we don't make eye contact. We don't shake hands or those things… If I start the conversation with, ‘Oh no, I don't shake hands,’ something like that, then people won't be open to me.

Similarly, some participants worked diligently at ridding themselves of accents, after it became evident that accented English undermined their professional credibility: “I had to work really hard to not have that accent. Because having that accent… if I sound a certain way, then I must not be as credible. Or I must not be as valuable.” This participant described considerable effort to assimilate, yet went on to say, “My value shouldn't be measured because I look Asian or I sound a certain way.” Another person attributed their professional success to having ‘non-accented’ English.

### Resilience and responding to racism

A common response to the ongoing, everyday nature of racism in nursing was to ‘rise above it’: “I’ve just developed a thicker skin and kind of let it roll off my back, even though it hurts a lot. It hurts that your colleagues think so poorly of you.” One participant said, like many racialized nurses she keeps her head down at work regarding racism: “I’ve never approached the person about it, just nowadays with the whole workplace bullying and all this… I would be afraid to approach her and be seen as the aggressor… It might turn into something bigger.” More than one participant mentioned that the person naming racism may be cast as a ‘bully’ if the person responsible for a racist comment or action becomes distressed. This draws on readily available and dismissive stereotypes: “We’re seen as ‘angry Black women.’”

Despite this, some participants chose to speak up, resist, refusing to let racism go unchecked: “I’m gonna set them straight. I’m not going to be rude or anything, but I’m also not going to be tolerant of ignorance.” As one person commented, speaking out allowed her to let it go: “The times when I’ve had the courage to speak up, I usually feel better about it. And then I don't go on thinking about it a very long time.”

In the context of institutional whiteness, assimilation is expected of racialized nurses, and becomes a survival strategy. As noted earlier, some participants worked hard to change their accents to reduce racism in their daily work. While they cannot change their ethno-racial identities, they could manage stigma by minimizing its impact. Others turned to education to increase their professional credentials, hoping to augment their professional credibility and authority, perhaps opening more employment options.

Several participants said finding or creating community with other racialized nurses, or colleagues further afield, was a main strategy to cope with everyday racism. Sometimes this was purely social support, sometimes more strategic.

We have some friendships on our unit that are based on the fact, on our colour… Like, a group of the Black nurses, we go out sometimes, and we go and eat or celebrate each other birthdays, baby showers, different things like that. Nothing formal, just like that. And then I belong to a Black nurses’ group on Facebook… It gives us a sense of identity, of belonging, people with similar beliefs. It's nice.

One participant worked in a large workplace where people of color made up less than 10% of staff. She described the value of connecting: “A few of us have banded together… and we have quite a bit of conversation between ourselves, about how do we strategize and be strategic about what it is we’re experiencing.” Some brought this approach to mentoring racialized students, aiming to improve future environments. Many suggested that the structural racism that shapes work experiences for racialized nurses needs to be disrupted at the point of recruitment and in nursing education.

## Discussion

Clearly our participants’ experiences were shaped not only by racism, but also by their intersecting social locations of gender, class, religion, and so on. Here, we focus on racism, not because the others are irrelevant but because racism deserves concentrated attention. Class advantage – arguably attained through nursing credentials – does not protect against racism. Moreover, while participants embodied other social locations, they spoke almost entirely about the impacts of racism. As Gillborn has argued, racism may hold “personal or autobiographical primacy” wherein it becomes “the dimension of the social world, of our lived reality, that we … foreground in making sense of our experiences” (2015, p. 284).

Undoubtedly not surprising for racialized readers, nurses in this study experienced racism at interpersonal, institutional and structural levels, all intricately connected (Nazroo et al., 2020). The complex interconnections, and the monolithic nature of white supremacy may be daunting for those seeking change. Yet it is not so distant that it is impossible to tackle; it is also part of everyday interactions, practices and policies. These ‘closer-to-hand’ instantiations of systemic power structures can be more immediately amenable to change, if there is political will.

Interpersonal racism was perhaps most obvious, fitting with pervasive perceptions in Canada that only aberrant individuals enact racism (Hilario et al., 2018). Direct and indirect derogatory comments by patients and coworkers, even physical altercations – these painful experiences hurt. The injury is amplified by non-response from coworkers and managers, leaving the racialized nurse isolated and struggling to manage racism alone. Assuming racialized nurses can act as translators, assigning them heavier workloads, withdrawing the collegial team support that is normally readily available in nursing – these replicate the experiences of racism documented decades ago in Canada (Calliste, 1996; Das Gupta, 1996; Etowa et al., 2009). They are instances wherein institutional and interpersonal racism coincide, particularly when individual experiences of racism receive no meaningful response from managers.

Study participants recounted instances of institutionalized racism stretching back to their nursing school years, including those who were recent graduates. This reiterates concerned raised by others (Calliste, 1996; Das Gupta, 1996; Etowa et al., 2009; Hilario et al., 2018; Modibo, 2004). When racialized managers and nurse leaders are few and far between (Bouabdillah et al., 2021; Iheduru-Anderson, 2020; Jefferies et al., 2018; Premji & Etowa, 2014), that reality embodies and exacerbates institutional racism. Lacking support from colleagues and managers, nurses in this study were left in the precarious situation of needing to be super-competent, and hyper-careful, never giving anyone a chance to find fault with their work (Etowa et al., 2009; Iheduru-Anderson et al., 2021). Because they are always visible, particularly when moving up the nursing hierarchy, they remained under constant surveillance (Calliste, 1996; Cottingham et al., 2018; Etowa et al., 2009; Modibo, 2004), while always under the cloud of prevalent perceptions of incompetence (Brathwaite, 2018).

This cultural doubt about capability – an aspect of structural racism – can be mobilized by managers or coworkers, targeting racialized nurses through heightened surveillance and reporting (Brathwaite, 2018). Again, this constitutes an intersection of structural, institutional and interpersonal racism. Similarly, the expectations of demeanor, comportment and social etiquette in the profession demand of racialized nurses that they adhere to or adopt the social norms of whiteness, the ways-of-being prized in nurses. From eye contact to accent, racialized nurses must expend energy navigating whether, when and how to assimilate to ostensibly race-neutral norms of ‘professionalism.’ Those are ideological dimensions of structural racism, wherein white ways of being are cast as superior, normal and professional.

Racialized nurses may be left to generate effective (or tolerable) responses. In the context of dominant ideologies that cast them as angry, threatening (Etowa et al., 2017), speaking up carries costs. Yet silence, too, is costly. It inflicts a wound on the soul, the heart. Not surprisingly, the most important strategy for survival identified by participants was finding or creating community, building strategic solidarity with others who would understand, even when the affronts remain unspoken.

### The role of silence and speech

Iheduru-Anderson et al. (2021) suggest a culture of silence plays a key role in perpetuating racism in nursing. They point out that racialized nurses are most often the champions of progressive change, thus the ones to initiate discussions of racism, yet “are often silenced by the dominant White group who, consciously or unconsciously, want to maintain the *status quo*” (p. 122). Sometimes silencing discussion about racism takes the form of demanding more/better/stronger evidence. The silence of racialized nurses reported by participants in this study is a strategy for survival in a hostile professional environment. The silence of white nurses has less justification.

While the harms of racism in nursing are borne by racialized nurses, the responsibility for change must not lie (solely) with them. If whiteness is written on the bones of the profession, widespread commitment and action are needed to effect change. Yet white people are uncomfortable speaking about racism, and uncomfortable with that discomfort, sliding quickly into shame, guilt, anxiety, fear, defensiveness and anger (DiAngelo, 2018). Those are difficult emotions, yet pale in comparison to the emotional labor demanded of racialized nurses (Cottingham et al., 2018).

White people, are often afraid to speak up regarding racism, afraid of ‘getting it wrong,’ saying the ‘wrong’ thing, or creating tension (Iheduru-Anderson et al., 2021). Perhaps, underlying all of that, afraid of being ‘race traitors,’ failing to show solidarity with the dominant group (DiAngelo, 2018). In the early 1980s Black lesbian feminist Audre Lorde reminded racialized people, “Your silence will not protect you,” noting that they would face anger, contempt, judgement, challenge, censure whether they spoke up or not (1984, p. 41). She asked, “What are the tyrannies you swallow day by day and attempt to make your own, until you will sicken and die of them, still in silence?” (p. 41). In the poem, “Litany for Survival” she wrote, “When we speak we are afraid our words will not be heard nor welcomed, but when we are silent we are still afraid. So it is better to speak, remembering we were never meant to survive” (1978).

Yet Lorde also wrote of the silences of white dominant-group members, the failures to speak when speech is necessary. She wrote about speaking even when afraid, especially when afraid. She urged us all to ask, “What’s the worst that will happen? Then push yourself a little further than you dare.” And though people may be dismissive, or angry, “the world won’t end.” The difference, of course, is silence can indeed protect white people from the travails of racism. Silence can mean not facing contempt, judgement, anger or challenge. Silence, though, also means complicity with racism. There is no neutral ground.

While disrupting the culture of silence in the profession (Iheduru-Anderson et al., 2021) is critical, the work of dismantling racism from the position of whiteness and privilege also requires deep listening, learning more about racism and white supremacy, welcoming the expertise and guidance of racialized people. “Practicing critical allyship” (Nixon, 2019) includes making space for other voices, silenced voices. It is the responsibility of white people (too) to interrogate the ways whiteness is built into the foundations and everyday practices of nursing, the work of transforming institutions.

Declarations of moral outrage regarding racism are not enough – they serve primarily to re-establish white innocence (Larocque et al., 2021). Statements of ‘zero tolerance’ for racism within institutions are meaningless, when the foundations of those institutions are mired in colonial racism (Cabrera et al., 2016). The need to move more racialized nurses into management and leadership positions is urgent, to begin disrupting pervasive institutional racism (Brathwaite, 2018; Iheduru-Anderson & Wahi, 2021), rather than relying on the next generation of nurses to bring about change.

### Recommendations for change

From a detailed review of the literature, Iheduru-Anderson et al. (2021) recommend individual actions (critical self-reflection; development of awareness and sensitivity; continuing professional development) that might help movement toward more effective discussions regarding racism in nursing, and more specific actions. White members of the nursing profession need to learn and teach how to recognize racism at all levels, need to learn and teach how to break silence around racism, need to learn and teach how to practice critical allyship (Nixon, 2019).

Recognizing individual changes do not alter institutional and structural racism, others suggest the need for careful scrutiny of institutional hiring and promotion practices, to ensure racialized nurses enter positions of leadership (Brathwaite, 2018; Waite & Nardi, 2019). Supports need to be in place for those hired and promoted, to ensure their success is not undermined. Mentorship, peer support, and community building among racialized nurses and students might help provide power in numbers (Bouabdillah et al., 2021). Building concrete supports for racialized nurses and for anti-racist practice into institutions’ strategic operations can help ensure accountability.

In medicine, Kristoffersson et al. (2021) have suggested the value of bystander training, helping white people to identify and respond to racism, disrupting the normative passive complicity with racism. This approach may hold promise. Minimally, nursing education must incorporate structural analysis of racism, helping future nurses understand how it is infused throughout society – and the profession – and how it can be undermined. This means moving from cultural competency education to anti-racist education (Cottingham et al., 2018; Iheduru-Anderson et al., 2021), in curricula and accreditation standards. At an even deeper level, commitment to anti-racism in nursing demands critical interrogation, if not dismantling, of the theories, systems, models, frameworks, institutions, and practices that structure the profession, identifying where the knowledge and practice bases of the profession are mired in whiteness.

## Limitations

Our study is limited by having a relatively small sample that is also heterogenous, including members of several racialized groups. Recruitment was discontinued when health professionals became overwhelmed with the COVID-19 pandemic. Nonetheless, we began to hear familiar narratives in the interviews, a hallmark of theoretical saturation. At the same time, the heterogenous sample adds strength, in that it allows exploration of racism across multiple racialized groups, encouraging a focus on the profession and white supremacy, rather than on individual experiences. Having conducted only one interview per person is also a limitation. Thie intent was to reduce participant burden, a major concern when interviewing busy professionals. In fact, getting even an hour of people's time was challenging, with some nurses having to curtail the interview to return to clinical duties.

Our conceptual framework of interpersonal, institutional and structural racism (Nazroo et al., 2020) is a heuristic device that both helps and hinders. It allows us to tease apart multiple levels of racism operating within nursing in Canada, examining their inter-penetrations, but it also suggests neater separations among them than is realistic. Moreover, like all analytic frameworks, it draws attention to some things and away from others. It does not, for example, allow us to explore the complexities of epistemic racism, what counts as knowledge and who counts as a credible knower in a profession dominated by white supremacy.

## Conclusion

Systemic racism operates through interconnected social patterns of power relations that privilege some and hinder others on the basis of racialization. This qualitative study suggests racialized nurses in Canada experience racism at all levels: interpersonal, institutional and structural. Those levels connect in mutually reinforcing intersections, causing ongoing harm to racialized nurses. Though they employ numerous coping strategies, including resistance, the costs are high. The profession, and particularly white nurses and nurse leaders, have an obligation to develop the skills to recognize and analyze racism at multiple levels, and to enact critical allyship (Nixon, 2019), working with racialized therapists to dismantle this system of power. Meanwhile, building community supports for racialized nurses and students within the profession, and ensuring racialized nurses advance into leadership positions are critical steps.
